# Ethical engagement with artificial intelligence in faculty research education: evaluation of the New Knowledge Help (NKH) approach

**DOI:** 10.1186/s12909-026-09405-2

**Published:** 2026-05-21

**Authors:** Noor-i-Kiran Naeem, Zil-e-Fatima Naeem, Asfandyar Anwer

**Affiliations:** 1 DecodingEdu, Gojra, Pakistan; 2 Medical Education, ABWA Medical College, Faisalabad, Pakistan; 3 Government City Hospital, Toba Tek Singh, Pakistan

**Keywords:** Artificial Intelligence, Faculty Development, Ethical Engagement, Instructional Design

## Abstract

**Background:**

Artificial intelligence (AI) offers new opportunities in healthcare research; however, faculty often lack structured training on its effective and ethical use. Existing faculty development initiatives frequently emphasise tool awareness rather than supporting ethical, reflective, and process-oriented engagement with AI. The New Knowledge Help (NKH) approach was developed as a pedagogical framework to support ethical engagement with AI in faculty research education and was operationalised using instructional design principles. This study evaluated the NKH approach using Kirkpatrick’s four-level framework.

**Methods:**

A blended programme of six face-to-face and two synchronous virtual workshops was conducted in three cities of Pakistan from November 2023 to March 2024. A total of 290 faculty members participated in NKH-guided workshops focused on ethical engagement with AI across the research process. The NKH approach was grounded in the Four-Component instructional design (4 C/ID) model and evaluated using the Kirkpatrick model across four levels: reaction, learning, behaviour, and results. Data were collected through post-workshop feedback, pre- and post-knowledge tests, and a three-month follow-up survey considering learning application and self-reported scholarly outputs.

**Results:**

The study revealed high levels of participants’ satisfaction with workshop content and delivery (Level 1: mean satisfaction scores 4.4–4.95/5; Satisfaction Index = 87.2%). There was a 22% learning gain in post-test knowledge as compared to pre-test results (Level 2, *p* < 0.05). At Level 3, 30% of participants reported full implementation of NKH-aligned practices, 25% partial implementation, while 45% reported they had not yet applied the skills in the three-month follow-up. At Level 4, 6.2% of faculty completed full manuscript writing using AI tools, while 44% reported partial ethical integration of AI-supported practices in writing, with a significant association (*p* = 0.04).

**Conclusion:**

The study concludes that the NKH approach supports the ethical engagement of faculty with AI tools in research, as demonstrated by enhanced satisfaction, knowledge gain, and early scholarly activities. The varied practices of faculty at level 3 and 4 suggest the need for institutional support, peer networking, and explicit guidance. The NKH approach offers a structured and pedagogically informed framework for fostering ethical engagement with AI in faculty training.

**Supplementary Information:**

The online version contains supplementary material available at 10.1186/s12909-026-09405-2.

## Introduction

Over time, artificial intelligence (AI) has emerged as an important tool for scholarly work in healthcare education and research [1]. Appropriate use of AI in research offers multiple advantages, including predictive modelling in healthcare using data analysis and pattern recognition in large datasets [2, 3]. However, to be used effectively in healthcare research and education, AI integration must extend beyond having various AI tools available. Integration requires faculty and researchers to possess adequate digital competence, informed judgement about AI outputs, and an understanding of approaches that support responsible and ethical use [4]. Without this preparedness, AI engagement risks becoming superficial, inconsistently applied across contexts, or vulnerable to ethical and methodological concerns that compromise the integrity of scholarly work.

Despite the growing interest in AI-enabled research, its adoption within faculty-led scholarly work and teaching remains uneven. Many teachers continue to use AI tools despite limited prior exposure and structured training, as opportunities for guided practice are currently limited [5–7]. In the absence of such support, the use of AI often remains fragmented and uncertain, rather than being purposefully integrated into the research workflow. These gaps not only influence the depth and quality of research work but also shape faculty confidence, scholarly productivity, and long-term professional development [8]. In this context, the use of AI in research is not merely technical, but rather developmental, reflective of wider disparities in faculty preparedness and mentorship across institutions.

Globally, faculty development programmes have begun to respond to the need for AI awareness [9]. Current programmes often introduce tools in isolation to emphasise general familiarity, rather than positioning AI as a general tool to be used across various phases of research from idea generation to research dissemination [5, 7, 10]. Additionally, these initiatives are rarely anchored in pedagogical principles and commonly overlook critical dimensions such as ethical responsibility, mitigation of algorithmic bias, responsible use of generative models and the risk of over-reliance on automated outputs [11]. Moreover, evaluation of faculty development programmes is often restricted to faculty satisfaction, with fewer studies examining behavioural change and long-term research productivity [12, 13]. As a result, the field continues to advance unevenly, with awareness increasing faster than preparedness.

Faculty development literature consistently demonstrates that educational interventions are more effective when grounded in clear pedagogical intent and evaluated beyond participants’ immediate responses [14, 15]. There remains a need for approaches that support ethical engagement with AI, guide faculty through the research process in a structured manner and allow meaningful evaluation of educational outcomes.

**Fig. 1:**
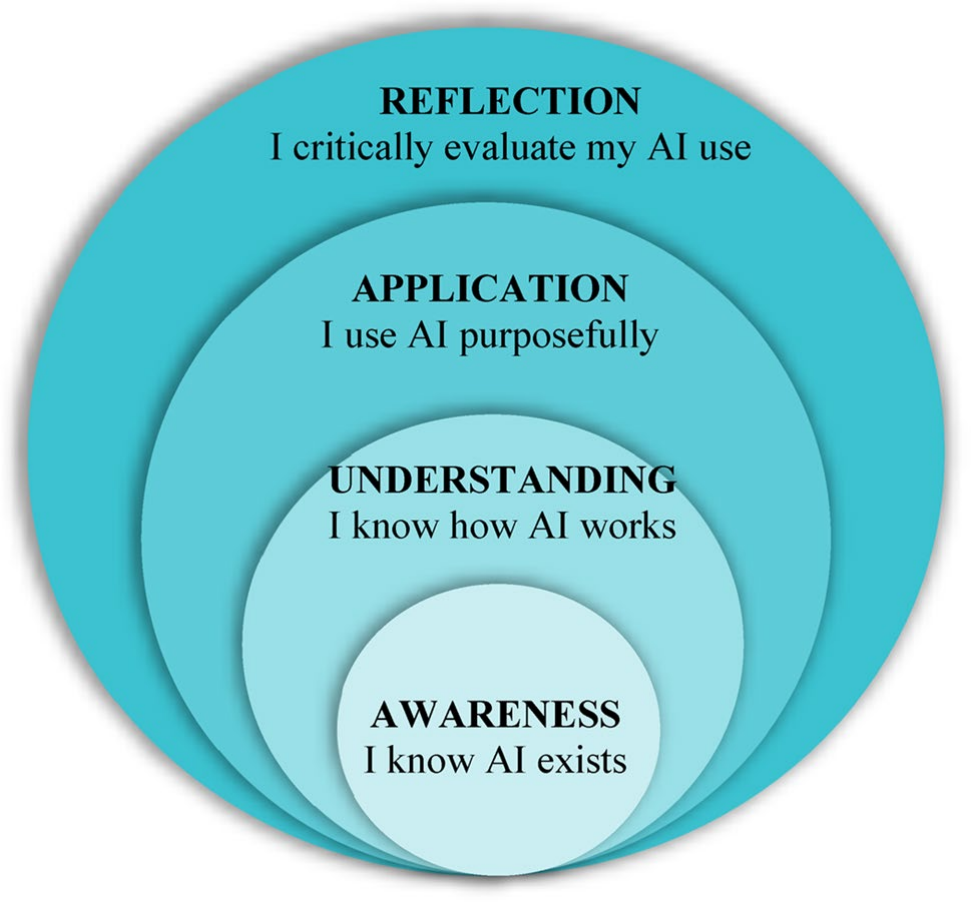
The NKH framework illustrating progressive stages of ethical engagement with artificial intelligence

In response to these gaps, the New Knowledge Help (NKH) approach was developed as a pedagogical framework to support ethical engagement with AI in faculty research education [16]. The NKH approach conceptualises AI engagement as a progressive learning continuum encompassing awareness, understanding, purposeful application, and critical reflection across the research process. Figure 1 illustrates the conceptual structure of the NKH approach.

The purpose of this study was to implement and evaluate the NKH approach as a scalable model for faculty research education. In doing so, the study examined how the approach supported faculty learning, shaped their experiences of engaging with AI in research tasks, and influenced early scholarly activities following workshop participation. The evaluation emphasised not only initial knowledge gains, but also the extent to which workshop participants applied AI-supported practices within their own research process. In this way, the study sought to generate early evidence of how a structured, pedagogically informed model may contribute to improved research confidence, ethical engagement, and emerging scholarly outcomes.

## Methodology

### Study design

This study employed a programme evaluation design to examine the implementation and outcomes of the NKH approach for ethical engagement with AI in faculty research education. A mixed-methods approach was used, analyzing both quantitative and qualitative data to evaluate educational outcomes during the study.

The evaluation was guided by Kirkpatrick’s four-level framework, assessing participants’ reactions to the intervention (Level 1), changes in knowledge and understanding (Level 2), transfer of learning into teaching and research practice (Level 3), and early scholarly outcomes (Level 4) [17]. Both formative and summative data were collected at predefined time points to capture immediate learning experiences, alongside short-term behavioural and scholarly outcomes.

### Study settings

The study was conducted from November 2023 to March 2024 across three major cities in Pakistan i.e., Lahore, Faisalabad, and Sialkot. Workshops were hosted at medical colleges and affiliated teaching hospitals and delivered using a blended format, comprising six face-to-face sessions and two synchronous virtual sessions.

Face-to-face workshops were conducted in computer laboratory settings, enabling participants to engage in guided, hands-on practice with AI tools. Virtual sessions were delivered using online platforms to reinforce learning, support continuity, and accommodate participants voluntarily joining from different institutions and locations.

### Study context

The study took place in a faculty research education context in a low-and middle-income country, where access to structured training in AI for research is variable. Participants represented diverse disciplinary backgrounds from basic and clinical sciences with differing levels of prior exposure to digital and AI-based research tools.

Institutional research support structures, including formal mentoring and protected research time, varied across participating institutions. While most participants were engaged in research and teaching supervision, many reported limited prior opportunities for structured, pedagogy-informed training in ethical and responsible AI use. This contextual variability informed the design and delivery of the NKH approach and was considered when interpreting the evaluation findings.

### Study participants and eligibility

A total of 290 faculty members from basic and clinical sciences participated in the eight workshops. Inclusion criteria were: (i) full-time or adjunct faculty with research supervision or teaching responsibilities, and (ii) the ability to attend at least one in-person and one virtual session. Exclusion criteria were: (i) prior completion of formal AI in research workshops, and (ii) not willing to consent to the study. Before enrolment in the study, informed consent was obtained from all the participants.

Participants represented a diverse academic community, varying in age, gender, academic position, and speciality. The majority of participants were female (53%) and in the 30–39 age range (48%). Most participants held Assistant Professor positions (52%) and were involved in the Basic Sciences (55%). The distribution of years in practice indicates a broad range of experience, with a large portion having less than 5 years in practice (32%). See Table 1 for the demographic distribution of the study participants.

**Table 1:** Demographic distribution of the study participants

Demographic Parameter	Frequency (*n* = 290)	Percentage (%)
Gender		
Male	137	47%
Female	153	53%
Age Range (Years)		
< 20	0	0%
20–29	17	6%
30–39	139	48%
40–49	87	30%
50+	47	16%
Academic Position		
Professor	38	13%
Associate Professor	102	35%
Assistant Professor	150	52%
Speciality		
Basic Sciences	160	55%
Clinical Sciences	130	45%
Years in Practice		
< 5	93	32%
5–10	58	20%
10–20	61	21%
20+	23	8%

### Needs assessment informing workshop planning

An initial needs identification phase was undertaken to inform the planning and structuring of the faculty development research education workshops. A comprehensive literature review identified existing barriers to research planning, execution, academic writing, and supervision in healthcare contexts [18–21]. Barriers included difficulty in the identification of research problems, formulating problem statements and manuscripts, limited institutional support, and a lack of effective feedback mechanisms.

To understand the contextual needs, three focus group discussions were conducted with post-graduate trainees from two institutions (See supplementary material for interview protocol). The focus group discussions aimed to explore perceived challenges during research projects. Each focus group discussion involved eight volunteer participants and lasted between 60 and 75 min. The discussions were audio-recorded after taking informed consent, transcribed and analysed for themes related to problems faced by researchers during their research projects.

Thematic analysis of the focus group discussions revealed participants’ low confidence in conducting research, and their limited access to structured guidance and resources. Participants highlighted challenges faced at various stages of research, including problem identification, methodology planning, manuscript writing, data analysis, and resource access. The insights gained from the literature review and focus group discussions were then used to inform the planning and sequencing of training workshops, ensuring alignment with learner contextual needs ( See Implementation of NKH Workshop).

### Training implementation

The NKH approach was implemented through a structured faculty development programme with workshop design and delivery guided by instructional design principles drawn from the Four-Component instructional design (4 C/ID) model [22]. In this study, the 4 C/ID model was used as an implementation scaffold to structure learning tasks, supportive information, procedural guidance, and opportunities for practice across the research process.

Outcomes of the NKH approach were evaluated using the Kirkpatrick framework to capture participants’ reactions (Level 1), learning (Level 2), behavioural change (Level 3), and early scholarly outcomes (Level 4). By aligning pedagogical intent with instructional design (4 C/ID) and evaluation (Kirkpatrick), the NKH approach was designed to support a contextually adaptable and ethically grounded pathway for responsible AI adoption in medical education.

**Fig. 2:**
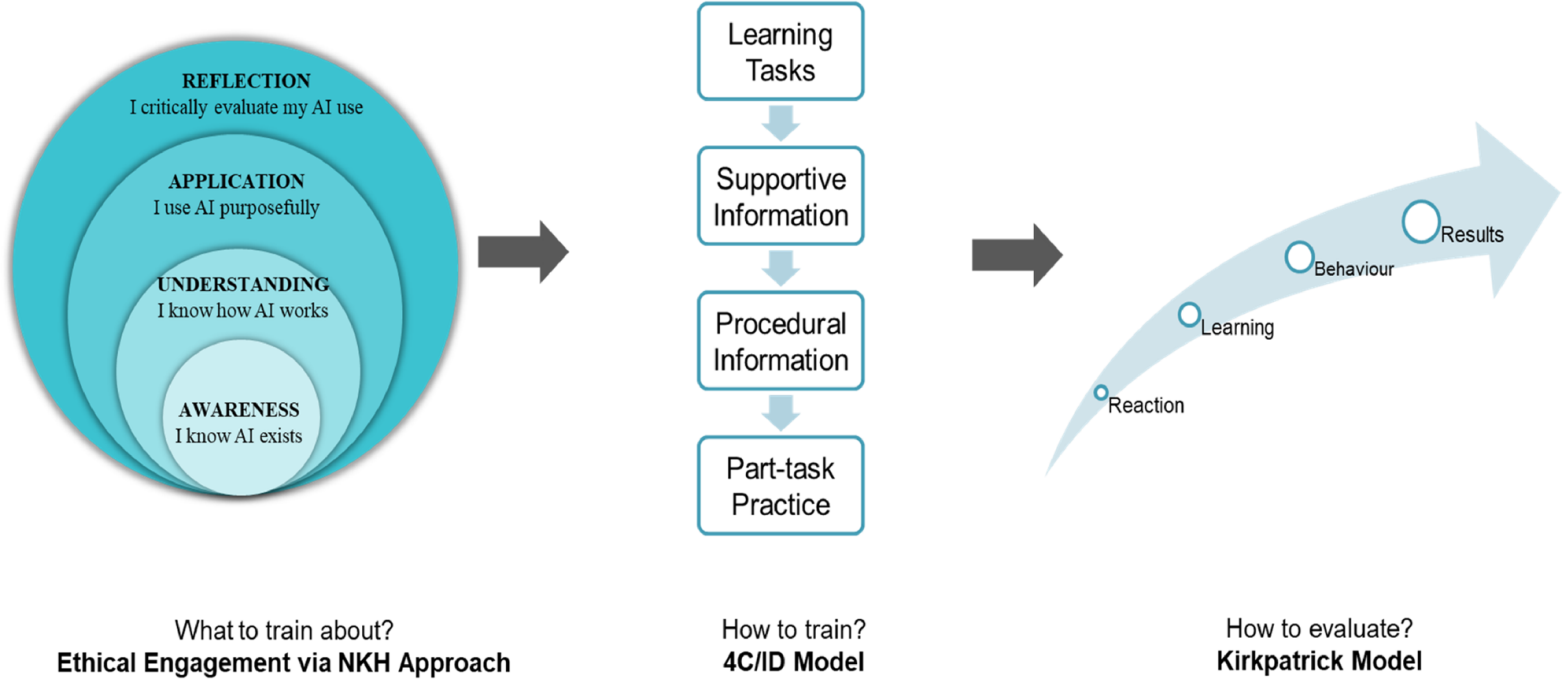
Conceptual framework linking the NKH approach, instructional design, and evaluation

Figure 2 illustrates the conceptual linkages between these three components, showing how the NKH approach defines a pedagogical progression operationalised through 4 C/ID-informed learning activities and evaluated across Kirkpatrick’s four levels.

## Implementation of the NKH Approach

The workshop followed a blended learning structure combining theoretical instruction with experiential hands-on activities, guided practice and reflective engagement. The content of the workshop was divided into the five key stages of the research process [23]:

1. Idea Generation: A demonstration was delivered followed by hands-on practice for using AI for brainstorming prompts, gap identification, and research question formulation using large language models. The NKH approach was employed to critically evaluate the AI-generated outputs.
2. Literature Review and Synthesis: This stage involved training regarding AI-supported search string refinement, summarising literature and identifying thematic patterns from selected AI tools. Participants engaged in comparing AI-assisted outputs with conventional approaches.
3. Research Design and Planning: Next, the participants explored use of AI tools to support research protocol development, instrument design, and data sample calculations.
4. Data Analysis: AI tools were introduced as supportive tools for data analysis rather than replacements. Various demonstrations included the use of initial qualitative coding assistance and descriptive analysis. Emphasis was placed on the researcher’s responsibility to ensure contextual interpretation of the results.
5. Manuscript Writing: The final stage focused on AI-assisted manuscript development with section structuring, language and grammar refinement and improving clarity. Participants practised short, guided tasks where AI-supported initial drafting but did not replace academic judgement.

Overall, the NKH approach prioritised process-oriented engagement over tool-specific instruction. The AI tools used for the demonstrations in the workshops are in Supplementary File 2.

### Data Collection

Data was collected via online Google forms corresponding to Kirkpatrick’s four levels of evaluation. These included participants’ satisfaction with the training (Level 1), learning gains after training collected via pre- and post-test results (Level 2 ), application of learning into practice (Level 3) and early scholarly outputs following implementation of the NKH approach (Level 4) [17]. The data for Levels 1 and 2 were collected after the workshops, and those for Levels 3 and 4 were collected three months after workshop completion.

### Level 1 (Reaction)

Participants’ reaction was requested through a feedback form on content relevance and clarity, facilitator effectiveness, practical usefulness, and confidence to apply AI tools. Responses were recorded on a five-point scale ranging from strongly disagree to strongly agree. This level captured participants’ satisfaction and perceived value of the learning experience.

### Level 2 (Learning)

Pre- and post-tests (15 items) measured knowledge of AI advantages, machine learning, natural language processing, and ethical safeguards. The pre-test was administered immediately before the intervention, and the post-test was administered at the conclusion of the workshop programme to assess changes in knowledge attributable to the intervention.

### Level 3 (Behaviour)

A structured follow-up survey administered three months after the post-training captured the extent of application in teaching and research practices. The survey assessed the extent to which participants had applied NKH-aligned practices in their academic work, including the use of AI-supported approaches for idea generation, literature synthesis, research design, data analysis, manuscript development, and peer support. The survey included Likert-Scale items and open-ended questions to capture both the frequency and nature of behavioural changes.

### Level 4 (Results)

Early scholarly outcomes were explored through self-reported indicators collected in a three-month follow-up. Participants reported outcomes such as AI-assisted manuscript planning or writing, peer education activities or mentoring related to AI-supported research practices and dissemination of learned practices within their institutions. These indicators have been used in prior faculty development literature to demonstrate scholarly productivity and knowledge transfer, which are expected outcomes of training programmes [24].

All instruments were reviewed by three invited medical education experts to ensure content relevance and clarity. Instruments were piloted with 15 faculty members during the pilot workshop to assess feasibility and comprehensibility. Data was checked for completeness and consistency before analysis.

### Data analysis

Quantitative data were analysed using SPSS version 26.0. Descriptive statistics, including means, standard deviations, frequencies, and percentages, were used to summarise participant responses. Paired t-tests were conducted to compare pre- and post-intervention knowledge scores. Chi-square tests were used to examine the association between training exposure and follow-up behavioural and scholarly outcomes. A p-value < 0.05 was taken as significant.

The qualitative data from focus group interviews and open-ended survey responses were subjected to inductive thematic analysis. Two coders independently developed codes, with an inter-coder agreement of 85%.

### Ethical considerations

The study was conducted in accordance with the ethical principles of the Declaration of Helsinki and established guidelines for educational research [25]. The study was approved by the Institutional Review Board of ABWA Medical College, Pakistan via letter no. MC/871/23 dated 24th October 2023. Participation was voluntary, and all participants received clear information about the study’s purpose, procedures, and use of data. Written informed consent was obtained before enrolment.

Participants were assured that participation or withdrawal would not affect their professional roles or evaluations. All data was anonymised prior to analysis, and no personally identifiable information was included in results. Data were stored securely with access restricted to the research team.

## Results

This study aimed to evaluate the NKH approach as a pedagogical framework for supporting ethical engagement with AI in faculty research education. The results are presented according to Kirkpatrick’s four-level evaluation model, describing participants’ reactions to the workshops (Level 1), changes in knowledge and understanding (Level 2), application of learned practices in research activities (Level 3), and early scholarly outcomes following the intervention (Level 4).

### Level 1: Reaction

Feedback collected immediately after the workshops revealed a high level of participant satisfaction. Participants rated the practical demonstration of AI applications in research (Mean = 4.8 ± 0.62) and facilitation quality (Mean = 4.8 ± 0.56). Content relevance received the highest score (Mean = 4.9 ± 0.74), while confidence in applying AI was rated lowest (Mean = 4.4 ± 0.56) ( Table 2).

**Table 2:** Level 1 Reaction Results

Level 1: Reaction	Mean Score (out of 5) (*n* = 290)
Effective practical demonstration of AI applications in research.	4.8 ± 0.62
Enhanced understanding of AI tools utilisation via hands-on activities.	4.7 ± 0.58
Workshop content is relevant and up to date.	4.9 ± 0.74
The facilitator was able to explain AI concepts and tools clearly.	4.8 ± 0.56
Participants’ confidence level in applying the AI tools in future research.	4.4 ± 0.56

**Fig. 3:**
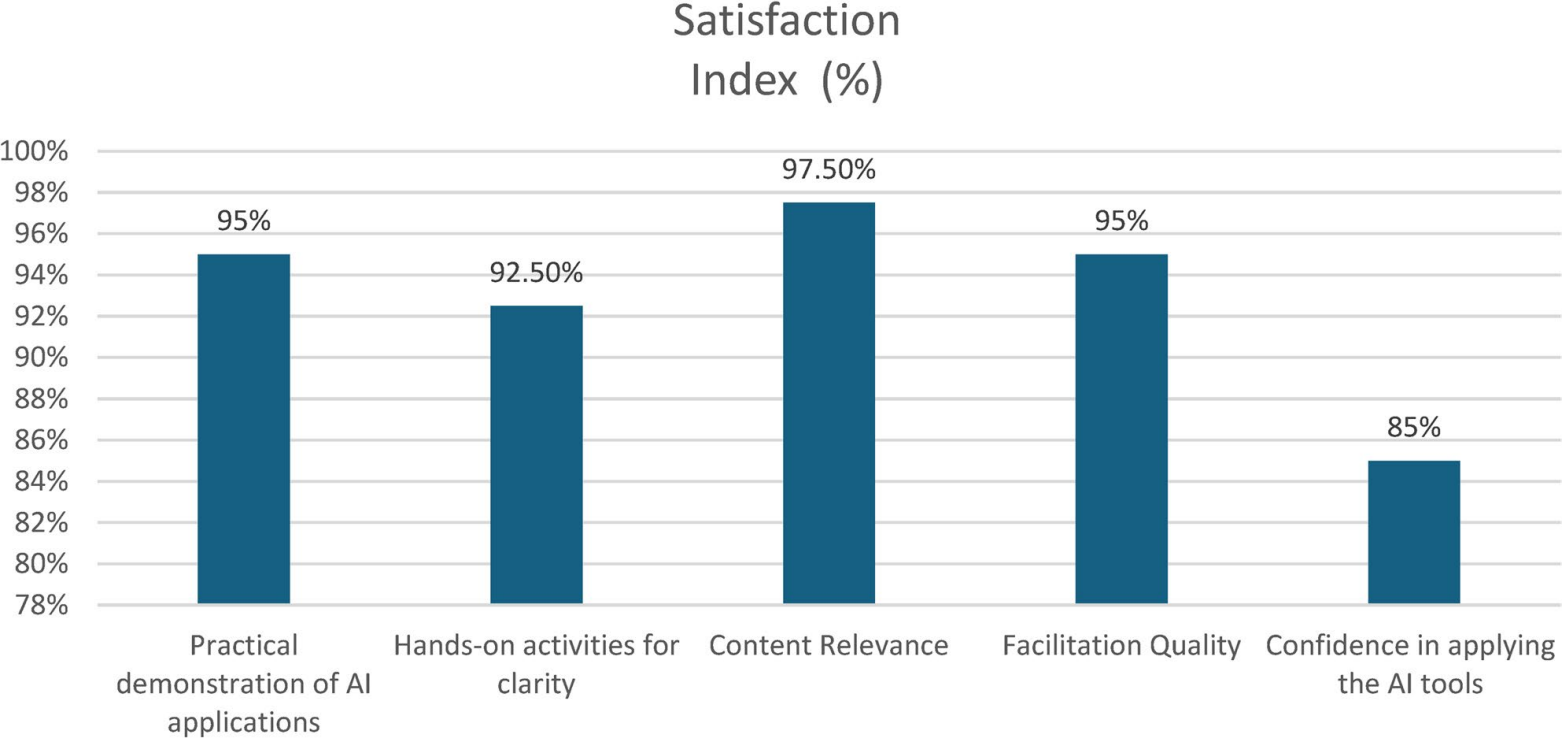
Satisfaction Index (%) in Level 1 (reaction results)

The Satisfaction Index (SI) across all items was 93.6%. Content relevance received the highest SI (97.5%), while confidence in applying AI tools received the lowest SI (85%) (Fig. 3).

### Level 2: Learning

Knowledge acquisition was evaluated through pre- and post-intervention assessments. Statistically significant differences were observed between pre- and post-intervention scores across all assessed domains. For example, participant understanding of the advantages of AI in medical research improved from a mean score of 1.2 ± 0.13 to 1.9 ± 0.21 (*p* < 0.0001). Statistically significant improvements were also observed for concepts such as machine learning, natural language processing, and ethical issues in AI research (Table 3).

**Table 3:** Level 2 Learning Gains Results

Level 2 : Learning Gains	Pre-test result (*n* = 290)	Post-test result (*n* = 290)	*p*-value
The primary advantage of using AI in medical research	1.2 ± 0.13	1.9 ± 0.21	0.0001
Comprehension of machine learning	0.8 ± 0.09	1.7 ± 0.11	
Understanding of natural language processing (NLP)	0.7 ± 0.10	1.6 ± 0.12	
Recognition of potential risks in AI research	0.9 ± 0.08	1.6 ± 0.12	
Awareness of ethical safeguards in AI research	1.1 ± 0.10	1.6 ± 0.11	

### Level 3: Behaviour

Three months post-intervention, participants were surveyed to assess their real-world application of the NKH approach. Full implementation was reported by 30% of the participants, 25% applied it partially, and 45% had not implemented it. There was a statistically significant association (*p* = 0.002) between workshop participation and post-workshop practical application. (Table 4).

**Table 4:** Level 3 Behaviour Results

Level 3: Impact	Not Done	Partially Done	Completely Done	*p*-value
Practical application of workshop knowledge	91 (45%)	50 (25%)	61 (30%)	0.002
Integration of AI tools and the NKH approach	71 (35%)	58 (29%)	73 (36%)	
Knowledge dissemination (teaching others)	91 (45%)	71 (35%)	40 (20%)	

**Fig. 4:**
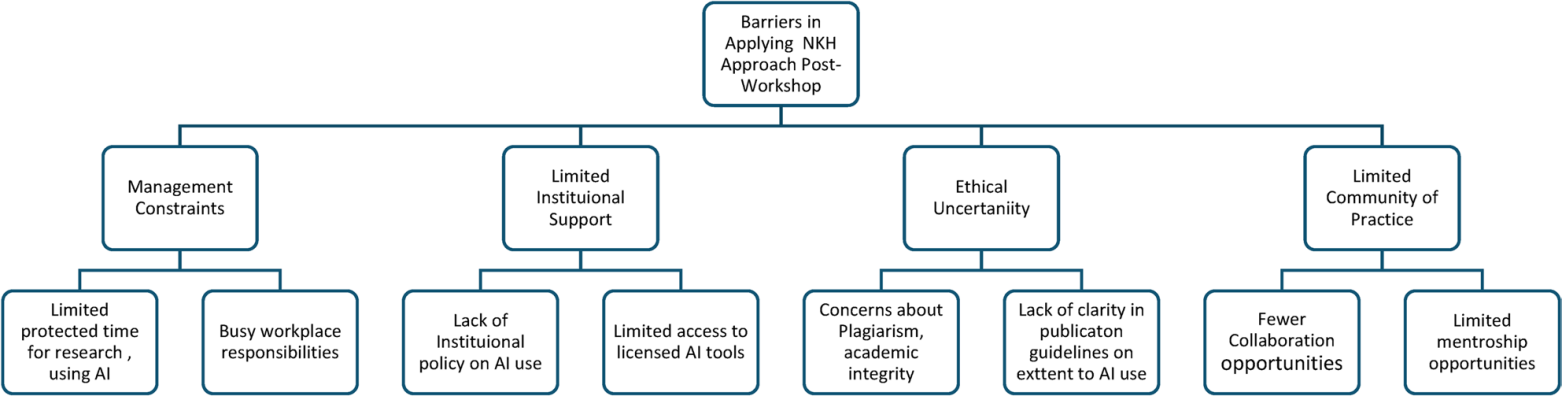
Identified barriers in applying the NKH approach post-workshop

Thematic analysis of open-ended responses from the participants who had not implemented NKH practices post-workshop (45%) revealed several barriers. The four categories of barriers were as follows: (i) management constraints, (ii) limited institutional support, (iii) ethical uncertainty, and (iv) lack of a community of practice. Each of these themes was supported by participant reflections, as summarised in Fig. 4.

### Level 4: Results

Following the workshops, 9% of participants reported fully integrating AI into manuscript writing, while 44% had done so partially. Following the workshop, 55% of participants reported providing training to others on AI tools. There was a statistically significant association between workshop participation and reported scholarly activities (*p* = 0.04) (Table 5).

**Table 5:** Level 4 Scholarly Outcome Results

Level 4: Scholarly Outcome Results	Not Done	Partially Done	Completely Done	*p*-value
Manuscript writing	95 (47%)	89 (44%)	18 (9%)	0.04
Educating others about AI tool use in research	71 (35%)	111 (55%)	20 (10%)	

## Discussion

This study aimed to evaluate the NKH approach as a pedagogical framework for integrating AI into the research process during training workshops. Results revealed positive participant reactions, significant knowledge gains, varying levels of practical application and early scholarly activities following the workshops. Using the NKH approach in faculty development workshops offers a structured approach for integrating AI into the research process, thereby contributing to emerging efforts to strengthen ethical engagement around AI use in Pakistan. International evidence suggests that well-designed faculty development initiatives can support teaching and research practices [26]. Consistent with programme evaluation design, these findings are interpreted as indicators for learning and early practice rather than definitive evidence of effectiveness.

Multiple studies have reported similar interventions demonstrating knowledge gains in AI literacy and digital capacity after training [27, 28]. However, these initiatives remain limited to short-term learning outcomes, limiting their impact on long-term scholarly practice [29, 30]. By linking instructional design with early scholarly activities, the NKH approach workshops address a documented gap in the evaluation of focused faculty development programmes rather than positioning themselves as a replacement for existing frameworks.

The use of AI tools varied across the five stages of the research process. Participants reported greater confidence in using AI tools for literature reviews and manuscript drafting. These patterns mirror international trends, where natural language processing and large language models are widely applied to academic writing and synthesis tasks [31]. By contrast, AI was less commonly applied to research design and problem framing. This observation is consistent with previous studies highlighting the limited role of AI in hypothesis generation and methodological planning [32]. It is also important to note that this study was conducted during an early phase of global AI adoption, and the range and maturity of available tools continue to evolve.

Not all the faculty participants transferred the learned practices into their teaching or research within the three-month follow-up period. Half of the participants reported no application of NKH-aligned practices during this period. Barriers identified included heavy workload, a lack of institutional support, ethical uncertainty, and the absence of a peer support system. These themes echo reports from low- and middle-income countries, where structural limitations reduce adoption [33, 34]. These findings highlight that individual training alone may be insufficient to ensure sustained behavioural change. To ensure sustained and comprehensive integration of AI tools into research, ongoing engagement and resources will be essential [30].

The observed variation in learning application further underscores the importance of ongoing scaffolding, mentorship, and institutional readiness [35–37]. Faculty members may require continued access to digital resources, peer collaboration networks, and structured follow-up opportunities to ensure meaningful integration of AI into their scholarly workflows [37]. Addressing these support mechanisms is likely to be critical for maintaining engagement and preventing widening disparities in AI literacy within academic environments.

Context also plays a crucial role. In Pakistan and other lower- and middle-income countries, uneven digital readiness, policy gaps, and limited infrastructure pose challenges [38]. However, opportunities exist to use AI tools to reduce time burdens, improve academic writing and provide new researchers with support that is otherwise scarce. As the NKH approach is process-oriented rather than tool-dependent, it may be adapted to diverse institutional contexts with varying levels of technological maturity.

Ethical considerations remain central to AI adoption in research education [30]. Faculty participants recognised the potential value of AI in improving manuscript quality, but expressed concerns about plagiarism, bias, and the acceptability of AI-generated texts in scholarly publishing. These concerns are widely noted in the international debate on responsible AI in research [39]. Accordingly, faculty development initiatives should address not only technical skills but also ethical reasoning, transparency, and alignment with institutional and journal policies.

The trustworthiness of these findings should be viewed in the light of the study design. All instruments were developed through expert review, piloted with faculty participants, and refined to ensure content validity. While these steps strengthen confidence in the findings, the outcomes, particularly manuscript development and peer education, remain self-reported indicators of early scholarly activities [6]. While the inclusion of a large multi-institutional sample enhances relevance within Pakistan, caution is warranted in extending these conclusions to other contexts without further study replication.

This study contributes to the literature by proposing and evaluating a structured, pedagogy-informed approach to AI integration in faculty research education. The NKH approach is not presented as a theory or as a replacement for existing digital literacy frameworks, but rather as a contextual pedagogical framing that emphasises ethical, reflective, and process-oriented engagement with AI. Its strength lies in its combined use of instructional design principles (4 C/ID) to structure learning activities and the Kirkpatrick framework to evaluate educational outcomes across multiple levels.

### Strengths and limitations

The study benefited from a large multi-institutional sample and the use of both quantitative and qualitative data. Alignment with established instructional and evaluation frameworks further strengthens methodological rigour. However, several limitations should be noted.

The follow-up was relatively short (three months), limiting conclusions about long-term impact. We did not name specific AI tools to avoid bias and ensure neutrality, but this reduces the details about the tools’ specific effectiveness. Additionally, qualitative findings relied on self-reported data and may reflect social desirability. Future research should extend follow-up, validate outputs externally, and examine institutional enablers that can support sustainable adoption.

### Implications for practice and policy

The NKH approach suggests that faculty development in AI can extend beyond awareness-raising towards structured engagement with research outcomes. For practice, this implies designing faculty development initiatives around authentic research tasks rather than isolated tool demonstrations. From a policy perspective, to sustain AI adoption, institutions should establish supportive structures, including clear ethical guidelines, access to digital infrastructure, and communities of practice. Such support is particularly important in low- and middle-income settings, where resource constraints may otherwise limit the benefits of AI-enabled innovation.

## Conclusion

This study suggests that the NKH approach provides a structured, pedagogically informed pathway for integrating AI into faculty research education. Participants reported positive learning experiences and knowledge gains, but variable practical application and use in early scholarly activities within a short follow-up period. By combining instructional design principles with multi-level evaluation, the NKH approach demonstrates feasibility and relevance with early signs of benefit rather than confirmed effectiveness. The findings indicate that faculty development in AI should move beyond tool demonstrations towards process-oriented and ethically grounded approaches. While adoption was not universal, the identified barriers highlight the importance of institutional support, policy alignment, and community building. In resource-constrained contexts, the NKH approach offers a potentially adaptable model for promoting responsible innovation in using AI tools for research.

## Supplementary Information

Supplementary Material 1.

Supplementary Material 2.

## Data Availability

All data generated or analyzed during this study are included in this published article and its supplementary files.

## Abbreviations

AI: Artificial Intelligence
FD: Faculty Development
FG: Focus Group
LMICs: Low- and Middle-Income Countries
LLMs: Large Language Models
NKH: New Knowledge Help
SD: Standard Deviation
SI: Satisfaction Index
4C/ID: Four-Component Instructional Design
