# Supplementary material for: Ethical engagement with artificial intelligence in faculty research education: evaluation of the New Knowledge Help (NKH) approach
Source: BMC Med Educ. 2026 May 21;26:1318. doi: 10.1186/s12909-026-09405-2 (PMC13474874; doi:10.1186/s12909-026-09405-2)

**Figure: AI tools used in training program utilising NKH Approach**

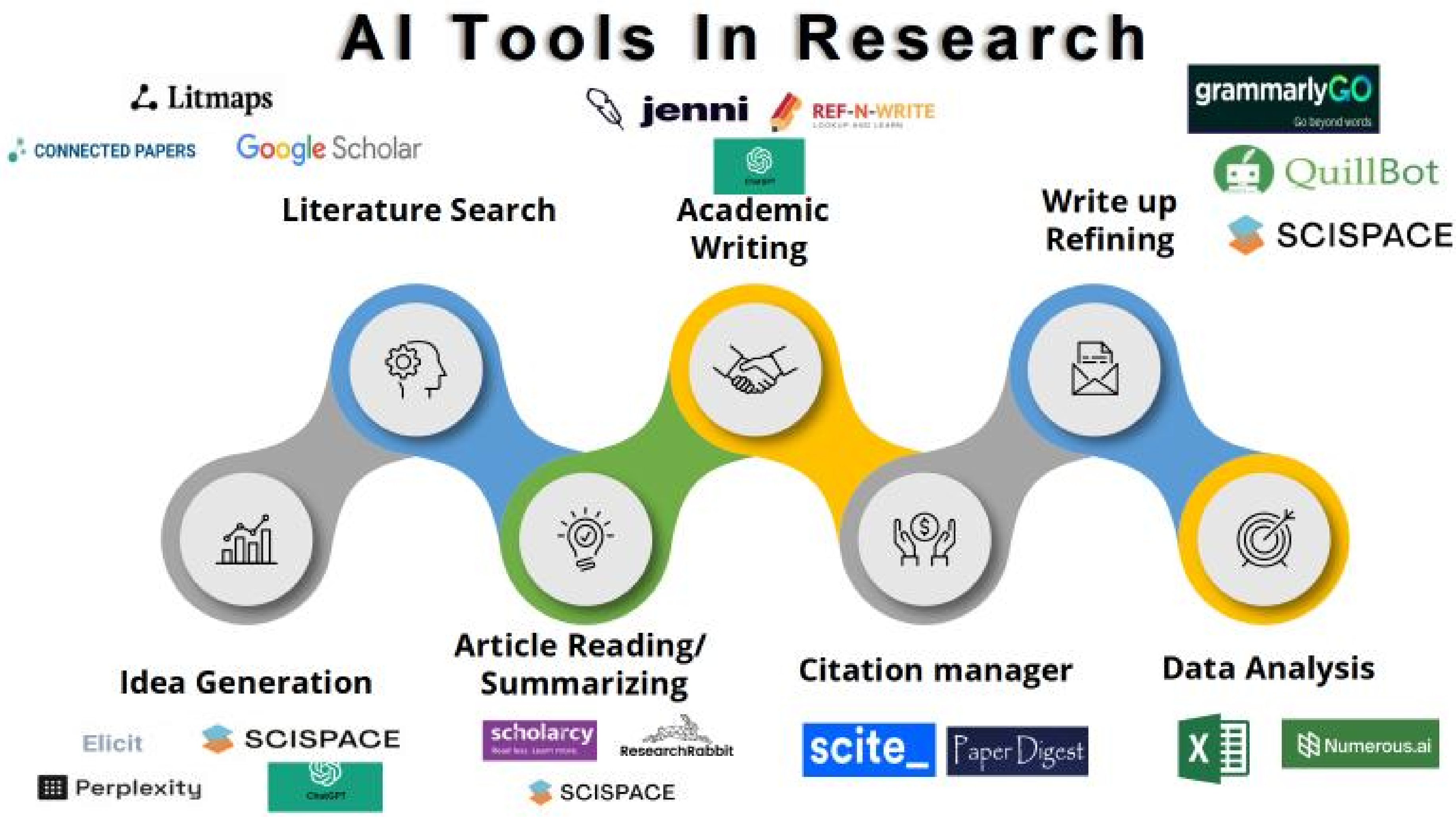

Supplement: Supplementary file 1 [file 12909_2026_9405_MOESM1_ESM.pdf]
