# Supplementary material for: Ethical engagement with artificial intelligence in faculty research education: evaluation of the New Knowledge Help (NKH) approach
Source: BMC Med Educ. 2026 May 21;26:1318. doi: 10.1186/s12909-026-09405-2 (PMC13474874; doi:10.1186/s12909-026-09405-2)
Supplement: Supplementary file 2 [file 12909_2026_9405_MOESM2_ESM.pdf]

Ethical Engagement with Artificial Intelligence in Faculty Research Education: Evaluation of the New Knowledge Help (NKH) Approach

Supplementary Interview Guide for Focus Group Interview

This semi-structured interview guide was developed specifically for the study titled '**Ethical Engagement with Artificial Intelligence in Faculty Research Education: Evaluation of the New Knowledge Help (NKH) Approach**' to explore the barriers and opportunities in integrating AI tools in healthcare research education from the perspective of postgraduate trainees.

Target Group:
Postgraduate medical trainees from two academic institutions in Punjab, Pakistan.

Interview Objectives:

1. To explore trainees' experiences and challenges in conducting healthcare research.
2. To identify gaps in digital literacy and AI-related competencies.
3. To inform the instructional design of the NKH (New Knowledge Help) approach.

Opening Questions:

1. Can you briefly describe your experience with conducting a research project during your training?

2. What kind of support or training have you received related to research methods and tools?

Main Discussion Areas:

3. What specific challenges have you faced during the planning or execution of research?

4. How confident do you feel in tasks such as writing a research proposal, conducting a literature review, or analyzing data?

5. Have you ever used digital or AI tools (e.g., referencing software, data analyzers, AI writing assistants) in your research? If yes, how?

6. What barriers do you see in accessing or using AI tools effectively?

7. How do you think AI could help improve the research process for medical trainees like yourself?

Closing Questions:

8. What kind of training or resources would be most helpful to support your use of AI in research?

9. Is there anything else you'd like to share about your experience with research education or AI integration?

Ethical Note:
All responses were anonymized. Participation was voluntary and part of an IRB-approved protocol at ABWA Medical College (Ref: MC/871/23).
